# Notes on the Composition of Scientific Papers

**Published:** 1905-03

**Authors:** 


**Notes on the Composition of Scientific Papers.** By T. CLIFFORD ALLBUTT, M.D., F.R.S. Pp. ix., 154. London: Macmillan & Co., Limited. 1904.

In the course of each year Professor Clifford Allbutt reads eighty to a hundred theses for the degrees of M.B. and M.D. Cambridge. He finds the greater number of these are badly composed. The composition is not merely inelegant, but such as "to obscure, to perplex, and even to hide or travesty the sense itself." In the little book before us the commoner errors and defects observed in such essays are discussed by the author in the hope of bringing about some reformation in composition for the future.

In the first or introductory chapter the difficulty of acquiring the art of writing well is appropriately emphasised. Even the greatest writers, it is pointed out, acquired their skill only after much laborious striving. The methods of composition are next dealt with, and here the manner of beginning and ending an essay, the selection of a title, the importance of summaries, the proper use of references, and the faults of eccentricity and finery in style receive due consideration.

In the remaining part of the book numerous examples of faults and errors in composition, taken largely from the theses mentioned above, are criticised and corrected. Counsel is given upon the choice and order of words, the avoidance of tautology and redundancy, the expression of emphasis, and the use of metaphors, quotations, and punctuation.

We cordially recommend this volume to the attention of all those writers of English who are not already masters of the art of writing the language correctly, lucidly, and gracefully, and we are sorry to have to admit that most of the scientific writing of the day is sadly deficient in these qualities. The great merits of the author's own composition as exhibited in these pages will be appreciated by all readers, and should promote the purposes he has had in view no less effectively than the precepts and examples he gives.

